# Glycogen Metabolism and Rheumatoid Arthritis: The Role of Glycogen Synthase 1 in Regulation of Synovial Inflammation *via* Blocking AMP-Activated Protein Kinase Activation

**DOI:** 10.3389/fimmu.2018.01714

**Published:** 2018-07-27

**Authors:** Maohua Shi, Jingnan Wang, Youjun Xiao, Cuicui Wang, Qian Qiu, Minxi Lao, Yangtao Yu, Zhifeng Li, Hongwei Zhang, Yujin Ye, Liuqin Liang, Xiuyan Yang, Guoqiang Chen, Hanshi Xu

**Affiliations:** ^1^Department of Rheumatology, The First Affiliated Hospital, Sun Yat-sen University, Guangzhou, Guangdong, China; ^2^Department of Rheumatology, The First People’s Hospital of Foshan, Foshan, Guangdong, China

**Keywords:** AMP-activated protein kinase, activation, fibroblast-like synoviocyte, glycogen, glycogen synthase 1, inflammation, rheumatoid arthritis

## Abstract

**Objective:**

To investigate the role of glycogen metabolism in regulating rheumatoid fibroblast-like synoviocyte (FLS)-mediated synovial inflammation and its underlying mechanism.

**Methods:**

FLSs were separated from synovial tissues (STs) obtained from rheumatoid arthritis (RA) patients. Glycogen content was determined by periodic acid Schiff staining. Protein expression was analyzed by Western blot or immunohistochemistry. Gene expression of cytokines and matrix metalloproteinases (MMPs) was evaluated by quantitative real-time PCR. FLS proliferation was detected by EdU incorporation. Migration and invasion were measured by Boyden chamber assay.

**Results:**

Glycogen levels and glycogen synthase 1 (GYS1) expression were significantly increased in the ST and FLSs of RA patients. TNF-α or hypoxia induced GYS1 expression and glycogen synthesis in RA FLSs. GYS1 knockdown by shRNA decreased the expression of IL-1β, IL-6, CCL-2, MMP-1, and MMP-9 and proliferation and migration by increasing AMP-activated protein kinase (AMPK) activity in RA FLS. AMPK inhibitor or knockdown AMPK could reverse the inhibitory effect of GYS1 knockdown on the inflammatory response in RA FLSs; however, an AMPK agonist blocked RA FLS activity. We further determined that hypoxia-inducible factor-1α mediates TNF-α- or hypoxia-induced GYS1 expression and glycogen levels. Local joint depletion of GYS1 or intraperitoneal administration with an AMPK agonist ameliorated the severity of arthritis in rats with collagen-induced arthritis.

**Conclusion:**

Our data demonstrate that GYS1-mediated glycogen accumulation contributes to FLS-mediated synovial inflammation in RA by blocking AMPK activation. In our knowledge, this is a first study linking glycogen metabolism to chronic inflammation. Inhibition of GYS1 might be a novel therapeutic strategy for chronic inflammatory arthritis, including RA.

## Introduction

Rheumatoid arthritis (RA) is a systemic chronic inflammatory disease characterized by the progressive destruction of cartilage and bone. Fibroblast-like synoviocytes (FLSs) in the synovial intimal lining are essential in the maintenance of synovial inflammation and progressive joint destruction (1, 2). Stable activated FLSs in RA exhibit tumor-like characteristics, such as anchorage-independent growth and resistance to apoptosis (3, 4), and play a crucial role in the development of pannus by migration and invasion toward cartilage and bone (5, 6). Evidence increasingly suggests that targeting FLS-mediated inflammation and invasion may have novel therapeutic potential for RA (7). However, the precise molecular mechanisms by which this pathogenic process is regulated are not clearly defined.

Glucose metabolism not only provides energy for physical activity but also modulates multiple physiological processes through the formation of complex signaling networks with metabolic substrates. Targeting the reprogramming of glucose metabolism has been considered as an attractive new approach to develop new therapeutics in cancer treatment (8, 9). Although some studies have suggested that aberrant glycolytic activity is associated with synovial tissue (ST) destruction and the autoimmune response in RA (8, 9), the role of glucose metabolism in the pathogenesis of RA has not received adequate attention.

In addition to glycolysis, glycogen metabolism has recently been proposed as an important pathway for metabolic reprogramming in some disorders and pathophysiological conditions (10–13). Glycogen is a form of glucose storage that is primarily present in liver and muscles. The synthesis and decomposition of glycogen are associated with the activity of several enzymes and regulatory proteins.

Glycogen synthase (GYS) is a rate-limiting enzyme in glycogen synthesis. GYS has two isoforms: glycogen synthase 1 (GYS1) and GYS2 (14). GYS1 is relatively ubiquitously expressed at lower levels in muscle, whereas GYS2 is primarily responsible for glycogen synthesis in the liver (15, 16). Increasing evidence suggests that glycogen turnover is altered in tumor cells. High levels of glycogen have been observed in several cancer cell lines (17). Glycogen accumulation is accelerated under hypoxic conditions in some types of cancer cells (11, 18–20). GYS1 and other proteins that control glycogen synthesis are also induced under hypoxic conditions in a hypoxia-inducible factor (HIF) 1α-dependent manner (10, 11, 20, 21).

The native form of AMP-activated protein kinase (AMPK), a critical modulator of energy balance (22), is a heterotrimeric complex that consists of a catalytic α subunit and accessory β and γ subunits. The AMPK-β subunit contains a centrally conserved region related to glycogen, which suggests that AMPK senses not only the immediate availability of cellular energy in the form of AMP and ATP but also the status of cellular energy reserves in the form of glycogen (23).

Previous reports have suggested that glycogen plays a key role in promoting the survival of tumor cells. For example, glycogen accumulation in cancer cells promotes survival in response to hypoxia and glucose deprivation (10, 11). Consistent with these observations, overexpression of GYS1 is associated with poor outcome in patients with acute myeloid leukemia (24, 25). These studies suggest an important role of glycogen metabolism in controlling tumor survival; however, it is still unclear if glycogen metabolism is associated with the regulation of chronic inflammation. Therefore, in this study, we investigated the relationship between glycogen metabolism and FLS-mediated synovial inflammation in RA.

## Materials and Methods

### Preparation of Human STs and FLSs

Synovial tissues were obtained from active RA patients (nine females and three males aged 40–65 years) or osteoarthritis (OA) patients (five females and three males aged 53–66 years) undergoing synovectomy or joint replacement. Healthy control (HC) STs were obtained by arthroscopic biopsy from five patients who had meniscus injuries or cruciate ligament rupture without history of acute or chronic arthritis. RA was diagnosed according to the 1987 ACR revised criteria (24, 25), and OA was diagnosed according to the ACR criteria (26). The study was approved by the Medical Ethical Committee of the First Affiliated Hospital, Sun Yat-sen University and was performed according to the recommendations of the Declaration of Helsinki. All patients provided written informed consent before participating in the study.

Synovial tissues were cut into small pieces and digested with 2 mg/ml collagenase type 1 (Life Technologies, Grand Island, NY, USA) for 3 h at 37°C to isolate synoviocytes. All cells were cultured in DMEM/F12 with 10% FBS (Life Technologies) at 37°C and 5% CO_2_. In our experiments, cells were used from passage 4 to 6, during which time they became a homogeneous population of cells (<1% CD11b positive, <1% phagocytic, and <1% FcgRII and FcgRIII receptor positive).

### Construction of Short Hairpin RNA-Expressing Lentivirus and Infection of FLSs

Lentivirus-based shRNA vectors were constructed as described previously (27). The sequences of the shRNA oligonucleotides are listed in Table S1 in Supplementary Material. Briefly, the shRNA-expression lentiviral vectors were generated by cloning gene-specific or scramble shRNA sequences into pLKO.3G vectors (Addgene, Cambridge, MA, USA), and lentiviruses were produced by co-transfection of HEK 293T cells with expression vectors and the helper plasmids pCMV-dR8.2-vprX and pCMV-VSVG using Lipofectamine 2000 (Life Technologies) according to the manufacturer’s protocol. Lentiviral particles were harvested from cell supernatants 48 and 72 h after transfection and purified by ultracentrifugation. The lentivirus was then determined. For infection, FLSs were infected with a 10% volume of concentrated lentiviral stock solution with 10 µg/ml polybrene (Santa Cruz Biotechnology, Dallas, TX, USA). The medium was replaced with fresh medium 8 h after infection. After 3 days of infection, gene expression and cell migration and invasion were analyzed. The transfection and infection efficiency were observed under an inverted fluorescent microscope.

### Immunohistochemistry

For immunohistochemistry, ST sections were deparaffinized, followed by incubation with 3% H_2_O_2_ for 10 min to block endogenous peroxidase activity and incubation with 5% serum in PBS for 30 min to block nonspecific binding. The expression of GYS1 was determined by staining with polyclonal rabbit anti-human GYS1 antibody overnight (Sigma-Aldrich, St. Louis, MO, USA) at 4°C. Irrelevant isotype-matched antibodies were used as controls. Polyclonal goat anti-rabbit antibodies labeled with horseradish peroxidase (Dako, Glostrup, Denmark) were used as secondary antibodies. Results were detected using diaminobenzidine.

### RNA Extraction and Quantitative Real-Time PCR (qRT-PCR)

Total RNA was isolated from FLSs using TRIzol reagent (Invitrogen, San Diego, CA, USA) according to the manufacturer’s protocol. Purified RNA samples were reverse transcribed with a PrimeScript™ RT reagent kit (Takara, Dalian, China) according to the manufacturer’s instructions. qRT-PCR was performed using the CFX384 Real-Time PCR Detection System (Bio-Rad, Hercules, CA, USA) and SYBR Green qPCR Master Mix (Takara). The primers employed for real-time PCR are listed in Table S2 in Supplementary Material. To quantify the relative expression of each gene, Ct values were normalized to the endogenous reference (ΔCt = Ct target − Ct 18S rRNA) and compared with a calibrator using the ΔΔCt method (ΔΔCt = ΔCt sample − ΔCt calibrator).

### Western Blot Analysis

Protein concentration was quantified using the BCA protein assay kit (Pierce Biotechnology, Rockford, IL, USA). Cell lysates were separated by electrophoresis in 10% acrylamide gels and transferred to nitrocellulose membranes (Millipore, Darmstadt, Germany). The membrane was probed with primary antibodies overnight at 4°C and subsequently with secondary antibodies (Invitrogen) for 1 h at room temperature. Proteins were visualized by enhanced chemiluminescence reagents (Promega Corporation, Madison, WI, USA) exposure to photographic film (Kodak). Western blot bands from several different blots were quantified using Image J software. The primary antibodies used were as follows: anti-GYS1 (Sigma-Aldrich), anti-phospho-glycogen synthase (Ser641, Cell Signaling Technology, Danvers, MA, USA), anti-AMPKα (Cell Signaling Technology), anti-Phospho-AMPKα (Thr172, Cell Signaling Technology), anti-HIF-1α (Abcam, Cambridge, UK), and anti-β-actin (Sigma-Aldrich).

### Glycogen Quantification

Glycogen levels in FLSs were measured using the Glycogen Colorimetric Assay Kit (BioVision, Milpitas, CA, USA) according to the manufacturer’s instructions, and results were normalized by protein content.

### Periodic Acid Schiff (PAS) Staining

The glycogen content of STs was detected by a standardized PAS staining technique using a PAS staining kit (Sigma-Aldrich). Briefly, sections were fixed with 4% paraformaldehyde for 15 min, incubated in 1% periodic acid for 5 min, rinsed with water, and incubated in Schiff’s reagent for 10 min. The slides were then washed with running tap water, counterstained with hematoxylin solution for 90 s, rinsed with water, dehydrated, cleared, and permanently mounted. Amylase (Sigma-Aldrich) was used to confirm that staining was specific for glycogen.

### *In Vitro* Migration and Invasion Assay of FLSs

Chemotaxis assays of FLSs were performed using the Boyden chamber method using a filter with a diameter of 6.5 mm and a pore size of 8.0 µm (Transwell; Corning Inc., Corning, NY, USA). Briefly, DMEM containing TNF-α (10 ng/ml, R&D Systems, Minneapolis, MN, USA) as a chemoattractant was placed in the lower wells. FLSs (at a final concentration of 6 × 10^4^ cells/ml) were suspended in serum-free DMEM in the upper wells. The chamber was incubated at 37°C under 5% CO_2_ for 8 h. After incubation, non-migrating cells were removed from the upper surface of the filter using a cotton swab. The filters were fixed in methanol for 15 min and stained with 0.1% crystal violet for 15 min. Chemotaxis was quantified by counting the stained cells that migrated to the lower side of the filter using an optical microscope (magnification 100×). The stained cells were counted as the mean number of cells per 10 random fields for each assay. For the *in vitro* invasion assay, similar experiments were performed using inserts coated with a Matrigel basement membrane matrix (BD Biosciences, Oxford, UK).

### FLS Proliferation Assays

Rheumatoid arthritis FLSs were cultured for 24 h at a density of 1 × 10^4^/well in 96-well plates in serum-free medium. After starving, the cells were incubated with 50 µM EdU for 8 h. EdU incorporation was assessed in triplicate using a Cell-Light™ EdU Apollo^®^488 *In Vitro* Imaging Kit (Ribobio, Guangzhou, China) according to the manufacturer’s instructions.

### Cloning of the Human GYS1 Gene Promoter and Luciferase Assay

Glycogen synthase 1 promoter fragments were amplified by PCR and inserted into the PGL3-basic vector (Promega Corporation) using SacI and NcoI (New England BioLabs, Ipswich, MA, USA) as restriction sites. The mutation of the HIF1α binding site was performed by site-directed mutagenesis. All constructs were verified by DNA sequencing.

MH7A cells derived from a human RA FLS cell line were seeded into 24-well plates to a confluence of approximately 50–60% on the day of transfection. The above luciferase reporter plasmids and HIF-1α overexpression plasmid were transfected using Lipofectamine 2000. Luciferase activity was measured by a Luciferase Assay System (Promega Corporation) in a Glomax fluorescence detector (Promega Corporation) according to the manufacturer’s instructions. All assays were conducted in triplicate and performed at least three times independently.

### Local Joint Depletion of GYS1 or Intraperitoneal Administration of AMPK Agonist in Rats with Collagen-Induced Arthritis (CIA)

The animal studies were authorized by the animal experimental ethics committee of the First Affiliated Hospital of Sun Yat-sen University. CIA was induced in male Sprague-Dawley (SD) rats by intradermal injection of 200 µg of bovine type II collagen (2 mg/ml, Chondrex, Redmond, WA, USA) emulsified at a1:1 ratio (vol/vol) in complete Freund’s adjuvant (4 mg/ml, Chondrex), followed by a booster 1 week later using bovine type II collagen emulsified at a 1:1 ratio (vol/vol) in incomplete Freund’s adjuvant (Chondrex). The rats were monitored daily for signs of arthritis, and arthritis severity was scored on a scale of 0–5, as described previously (28). The total score was recorded as the sum of the scores in the four limbs. For local joint depletion of GYS1, 50 µl of recombinant lentivirus (Scramble or shGYS1, 5 × 10^8^ TU/mL) was injected per site in the ankle joints and hind paws on days 9 and 11 after the first immunization (days 9 and 11). To determine the effect of the AMPK agonist, rats were treated with an intraperitoneal injection of 5% DMSO (vehicle, once daily) or the AMPK agonist AICAR (25 mg/kg/day, once daily, Selleckchem, Houston, TX, USA) for a total of 14 days, initiated on the day of arthritis onset. After rat sacrifice and skin removal, the whole hind and fore limbs were fixed, decalcified, and embedded. Sections (5 µm) were stained with hematoxylin and eosin. Synovial infiltration, cartilage erosion, and bone loss were scored as described previously (28). All scoring was performed by two blinded pathologists.

### Statistical Analysis

Data analysis was performed using GraphPad Prism 5.0 software (GraphPad Software, La Jolla, CA, USA). Data are expressed as the mean ± SD. Student’s *t*-test or ANOVA was used to evaluate differences between experimental groups. *P* values less than 0.05 were considered statistically significant.

## Results

### Glycogen Synthesis and GYS1 Expression Are Increased in ST and FLSs From RA Patients

**Figure 1:**
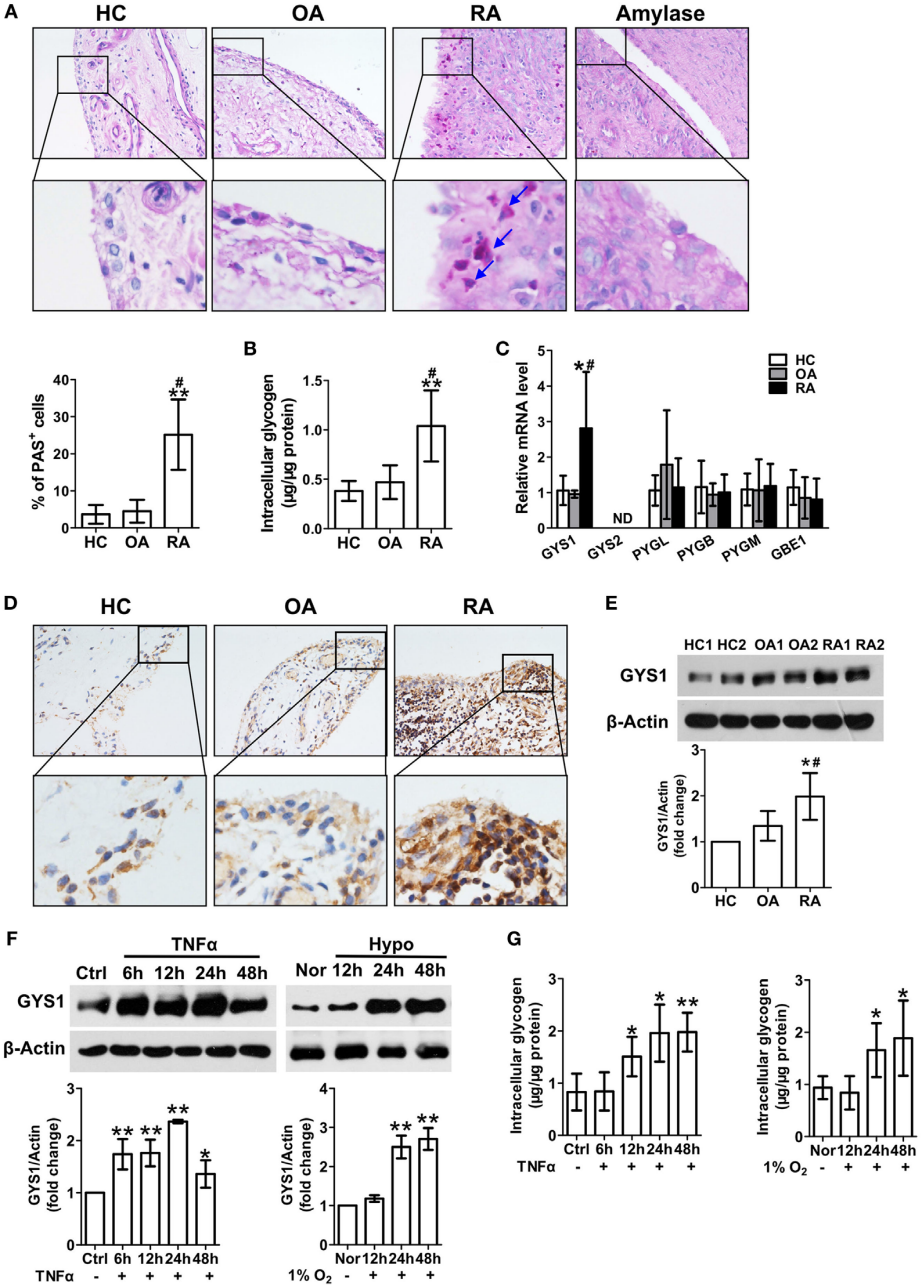
Increased glycogen synthase 1 (GYS1)-mediated glycogen synthesis in synovial tissue (ST) and fibroblast-like synoviocytes (FLSs) from rheumatoid arthritis (RA) patients. **(A)** Glycogen levels in STs from patients with healthy controls (HCs), osteoarthritis (OA), RA, confirmed to be glycogen by the absence of staining after amylase pretreatment. Representative images (upper panel) and semi-quantitative chart (lower panel) of periodic acid Schiff (PAS) staining for ST sections from HC (*n* = 5) and patients with OA (*n* = 6) and RA (*n* = 10). **(B)** Glycogen content in FLSs from HC (*n* = 5) and patients with OA (n = 6) and RA (*n* = 11). **(C)** Detection of glycogen metabolism-related gene transcripts by quantitative real-time PCR in FLSs from HC (*n* = 5) and patients with OA (*n* = 8) and RA (*n* = 12). **(D)** GYS1 protein expression in STs. Representative images of immunohistochemical staining of tissues from HC (*n* = 5) and patients with OA (*n* = 6) and RA (*n* = 8). Original magnification 200×. **(E)** GYS1 protein expression in FLSs. Representative images of immune blot (upper panel) and densitometric quantification (mean ± SD, lower panel) of GYS1 expression were from HC (*n* = 5) and patients with OA (*n* = 6) and RA (*n* = 12). **(F,G)** FLSs were exposed to TNF-α (10 ng/ml) or hypoxia (hypo, 1% O_2_). GYS1 expression **(F)** was detected by Western blot. The densitometric quantification [lower panel of **(F)**] of immune blot and glycogen levels **(G)** is presented as the mean ± SD of four independent experiments. *P < 0.05, **P < 0.01 vs. HC or normoxia (nor) or Ctrl; ^#^*P* < 0.05 vs. OA.

Glycogen content, as determined by PAS staining, was increased in STs from RA patients compared with HCs and OA patients (Figure 1A). There were no PAS staining in the STs from RA patients treated with amylase. Glycogen levels were also elevated in FLSs isolated and cultured from RA patients compared with HC and OA FLSs (Figure 1B). These results suggest increased glycogen accumulation in the RA synovial membrane.

To identify the factor that controls glycogen accumulation, we further analyzed glycogen metabolism-related gene transcripts in FLSs. As shown in Figure 1C, among all glycogen metabolism-related genes, only GYS1 gene expression was higher in RA FLSs than in HC FLSs and OA FLSs, indicating that GYS1 may be critical in regulating abnormal glycogen accumulation in the synovial membrane in RA patients. Therefore, we further determined the expression of the GYS1 protein in STs and FLSs. Consistent with our gene expression data, GYS1 protein expression was prominent in STs from RA patients and was mostly localized in the synovial lining and sublining cells. The expression of the GYS1 protein was less prominent in STs from HC and OA patients (Figure 1D). Western blotting analysis further confirmed that GYS1 protein expression was higher in RA FLSs than in HC and OA FLSs (Figure 1E). Because hypoxia and pro-inflammatory cytokines, particularly TNF-α, play important roles in synovial inflammation and joint destruction, we observed the effect of TNF-α or hypoxia on GYS1 expression in confluent RA FLSs. Treatment with TNF-α (10 mg/ml) or hypoxia (1% O_2_) increased GYS1 expression (Figure 1F) and glycogen levels (Figure 1G) in RA FLSs.

In addition, because glucocorticoids and methotrexate (MTX) therapy are central to current treatment paradigms for RA, we observed the effect of dexamethasone (DXM) and MTX on GYS1 expression and glycogen synthesis in cultured RA FLSs. Treatment with DXM or MTX reduced GYS1 expression (Figure S1A in Supplementary Material) and intracellular glycogen levels (Figure S1B in Supplementary Material). Taken together, these results suggest that abnormal GYS1-mediated synovial glycogen accumulation may be involved in the pathogenesis of RA.

### GYS1 Knockdown Decreases the Expression of Pro-Inflammatory Cytokines and Matrix Metalloproteinases (MMPs) and the Proliferation and Migration of RA FLSs

**Figure 2:**
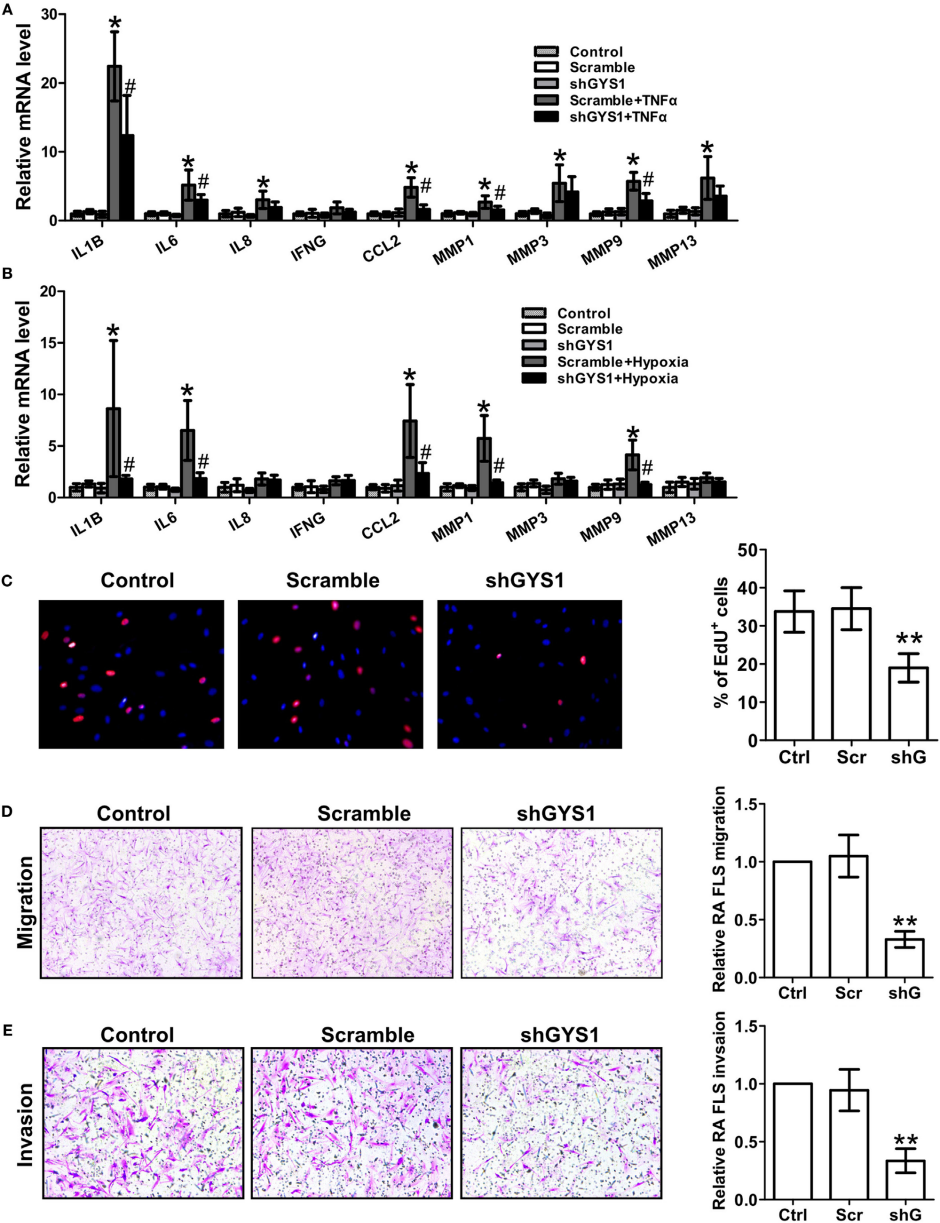
Effect of glycogen synthase 1 (GYS1) knockdown on rheumatoid arthritis (RA) fibroblast-like synoviocyte (FLS) function. shGYS1-2 was used in the subsequent experiments. **(A,B)** Effect of GYS1 knockdown on pro-inflammatory cytokines and matrix metalloproteinase (MMP) expression induced by TNF-α **(A)** or hypoxia **(B)**. Transfected FLSs were unstimulated or stimulated with TNF-α (10 ng/ml) or cultured under normoxic (21% O_2_) or hypoxic conditions (1% O_2_) for 24 h. mRNA expression was determined by qRT-PCR. The data are presented as the mean ± SD of five independent experiments. **(C)** Cell proliferation was determined by EdU assay. Data are presented as the mean ± SD of five independent experiments (right panel). Original magnification 400×. **(D,E)** Effect of GYS1 knockdown on the migration **(D)** and invasion **(E)** of RA FLSs. Cells transfected with GYS1 shRNA (ShG) or scramble (Scr) were serum starved overnight, seeded in a Boyden chamber, allowed to migrate for 8 h, fixed, and stained with crystal violet. TNF-α was used as a chemoattractant. An *in vitro* invasion assay was performed using inserts coated with a Matrigel basement membrane matrix in Boyden chambers. The data are presented as the mean ± SD of five independent experiments. The images are representative of migration or invasion through the membrane after staining. Original magnification 200×. *P < 0.05, **P < 0.01 vs. scramble; ^#^*P* < 0.05 vs. scramble + TNF-α or scramble + hypoxia.

To determine the role of GYS1-mediated glycogen synthesis in the synovial inflammation of RA, we utilized RNA interference to selectively reduce GYS1 expression. To rule out nonspecific interference, we constructed three different sequences of shRNA oligonucleotides for GYS1. As shown in Figures S2A,B in Supplementary Material, transfection with all three shRNAs downregulated GYS1 protein expression and intracellular glycogen levels. After 72 h of transfection, the RA FLSs were treated with TNF-α for 24 h. Treatment with GYS1 shRNA resulted in decreases in the expression of IL-1β, IL-6, and CCL-2 but not IL-8 and IFN-γ in TNF-α-induced RA FLSs compared with the scramble control (Figure 2A; Figure S2C in Supplementary Material). GYS1 shRNA transfection also decreased the TNF-α-induced expression of MMP-1 and MMP-9 but not MMP-3 and MMP-13 (Figure 2A; Figure S2C in Supplementary Material). Similar results were obtained in RA FLSs under hypoxic conditions (Figure 2B; Figure S2D in Supplementary Material).

Previous studies have demonstrated that GYS1 is involved in tumor cell proliferation. To determine if GYS1 regulates the proliferation of RA FLSs, the cells were treated with EdU (50 µM). As shown in Figure 2C; Figure S2E in Supplementary Material, GYS1 knockdown decreased the proliferation of RA FLSs. Furthermore, GYS1 shRNA transfection decreased the chemotactic migration of RA FLSs compared with the scramble control (Figure 2D; Figure S2F in Supplementary Material). We also observed an inhibitory effect of GYS1 knockdown on the *in vitro* invasion of RA FLSs (Figure 2E; Figure S2G in Supplementary Material). Collectively, these results suggest that GYS1-mediated glycogen accumulation may contribute to synovial inflammation and the invasive behavior of RA FLSs.

### GYS1 Knockdown Increases AMPK Activity in RA FLSs

**Figure 3:**
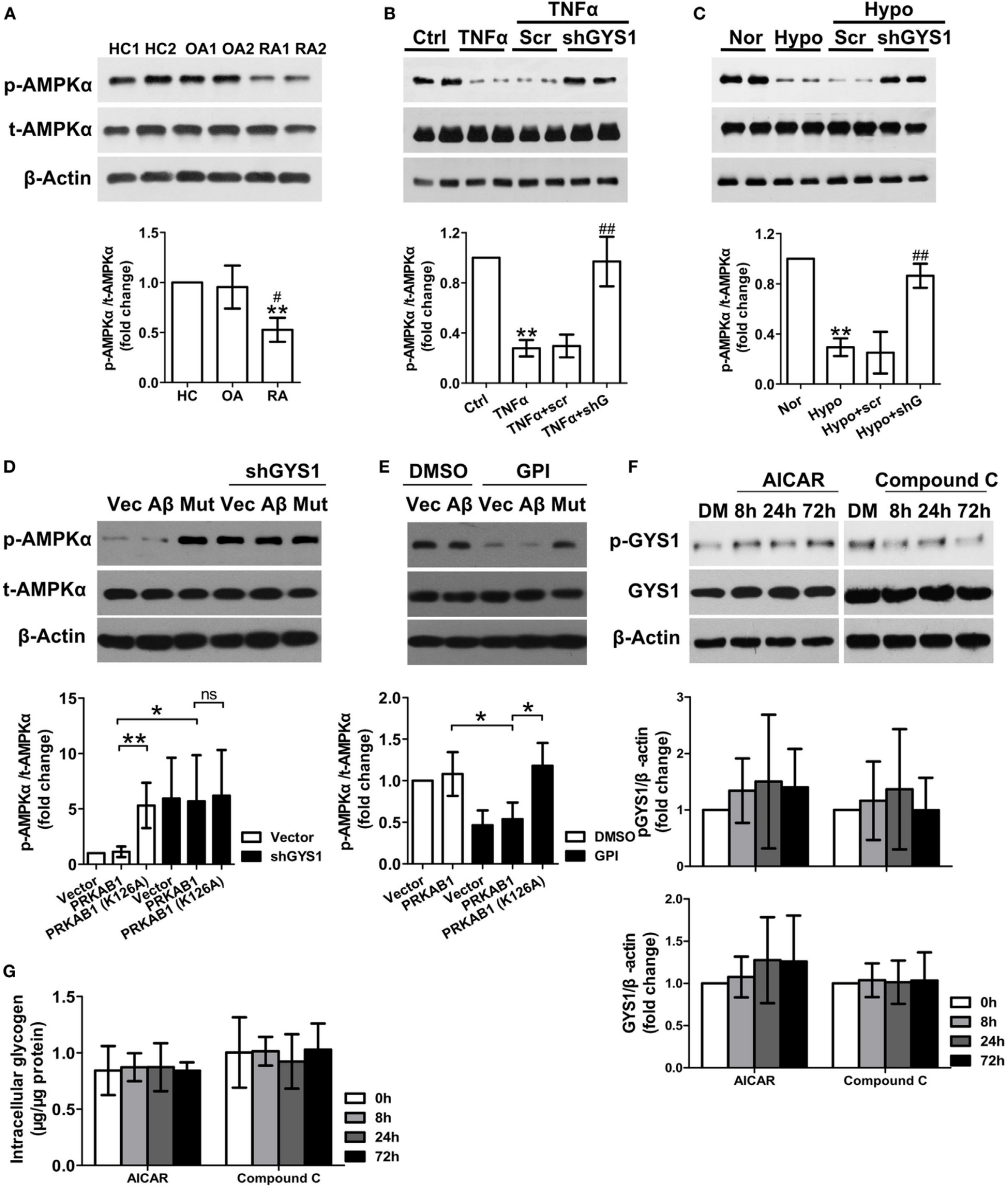
Effect of glycogen synthase 1 (GYS1) knockdown on AMP-activated protein kinase (AMPK) activity in rheumatoid arthritis (RA) fibroblast-like synoviocytes (FLSs). **(A)** AMPK activity in FLSs from healthy control (HC) (*n* = 5) and patients with osteoarthritis (OA) (*n* = 5) and RA (*n* = 9). Representative images of immune blots are shown (upper panel), and data from densitometric quantification (lower panel) are expressed as the mean ± SD. **(B,C)** Effect of GYS1 knockdown on AMPK activation induced by TNF-α **(B)** or hypoxia **(C)**. Transfected FLSs were untreated or treated with TNF-α (10 ng/ml) or cultured under normoxic (nor, 21% O_2_) or hypoxic conditions (hypo, 1% O_2_) for 24 h. The data represent the densitometric quantification (mean ± SD, lower panel) of four independent experiments. **(D)** Effect of AMPKβ1 (Aβ) and AMPKβ1 mutation (Mut) on AMPK activation induced by GYS1 knockdown **(D)** and glycogen phosphorylase inhibitor (GPI) **(E)**. **(F,G)** Effect of the AMPK agonist AICAR (AI) or inhibitor Compound C (Co) on phosphorylated and total GYS1 expression **(F)** and glycogen level **(G)** in RA FLSs at different time point. RA FLS were treated with AICAR (1 mM), Compound C (Co) (1 µM) or DMSO (DM) for different times (8, 24, and 72 h). The data are presented as the mean ± SD of four independent experiments. *P < 0.05 vs. HC or nor or control; ^#^*P* < 0.05 vs. OA or TNF-α + scr or hypo + scr.

The AMPK β subunits contain a central conserved region related to glycogen; therefore, to explore the mechanisms by which GYS1-mediated glycogen accumulation regulates synovial inflammation and invasion, we evaluated the relationship between GYS1 and AMPK in RA FLSs. AMPK activity but not total AMPK protein was lower in RA FLSs than in HC FLSs and OA FLSs (Figure 3A). Next, we determined the effect of GYS1 knockdown on AMPK activity. As shown in Figure 3B, treatment with TNF-α decreased the expression of phosphorylated AMPKα. However, this decrease was reversed by transfection with GYS1 shRNA. Similar results were obtained in hypoxia-treated RA FLSs (Figure 3C). In addition, treatment with DXM or MTX increased AMPK activity in RA FLSs (Figure S3 in Supplementary Material), in contrast to their effect on GYS1 expression, as shown in Figure S1A in Supplementary Material. These data suggest an important role of GYS1 in regulating AMPK activity in RA FLSs.

There are two isoforms of AMPK beta subunit, β1 and β2. We detected the expression levels of AMPKβ1 (PRKAB1) and AMPKβ2 (PRKAB2) in FLSs by qRT-PCR, however, we observed that the expression of PRKAB2 was absent in FLSs from HC, OA, and RA (Figure S4 in Supplementary Material). Therefore, AMPKβ1 was used a target to regulate AMPK activity in our experiment. To determine the direct interaction between glycogen and AMPKβ, we constructed a mutated PRKAB1 vector lacking the glycogen-binding site, in which the 126th Lysine of the protein was replaced with Alanine, and then determined the effect of AMPKβ1 mutation on AMPK activation induced by GYS1 knockdown (Figure 3D) and glycogen phosphorylase inhibitor (GPI) (Figure 3E). We observed that both PRKAB1 mutation and GYS1 knockdown increased the expression of phosphorylated AMPKα. Furthermore, GPI decreased the expression of phosphorylated AMPKα, it was reversed by transfection with mutated PRKAB1 vector. Therefore, these data suggest glycogen directly inhibits the enzyme activity of AMPK by binding to AMPKβ. Because the AMPK pathway has also been reported to be involved in glycogenesis by regulating GYS1 phosphorylation and GYS1 expression in tumor cells (25), we evaluated whether AMPK also modulates GYS1-mediated glycogenesis in RA FLSs. Interestingly, treatment with the AMPK agonist AICAR or inhibitor Compound C did not affect GYS1 phosphorylation and expression, intracellular glycogen levels at different time point (Figures 3F,G), suggesting that the AMPK pathway does not participate in GYS1-mediated glycogen synthesis in RA FLSs.

### The AMPK Pathway Regulates the Expression of Pro-Inflammatory Cytokines and MMPs and Proliferation and Migration in RA FLSs

**Figure 4:**
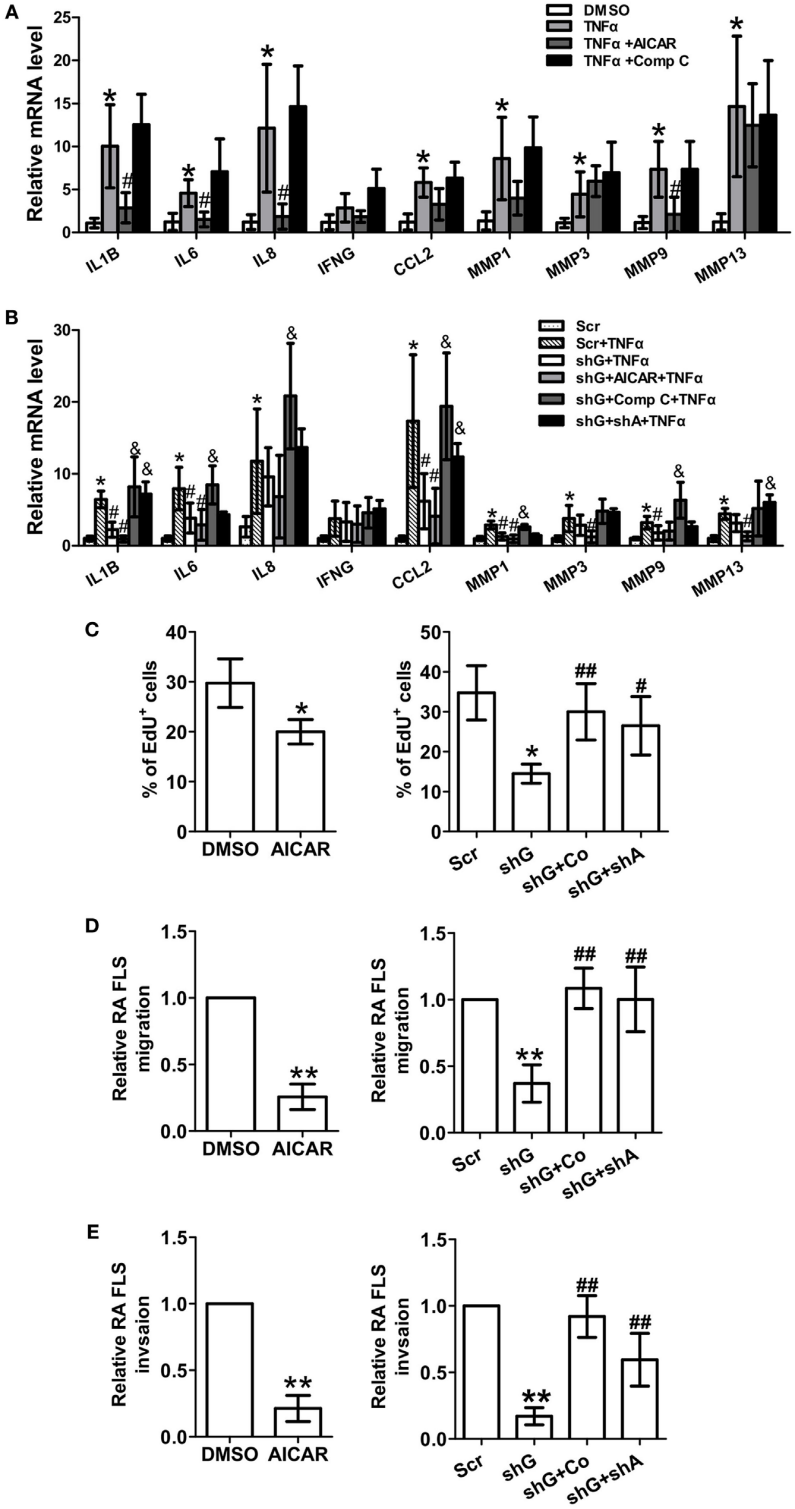
Effect of changes in AMP-activated protein kinase (AMPK) activity on rheumatoid arthritis (RA) fibroblast-like synoviocyte (FLS) function. **(A)** Effect of the AMPK agonist and inhibitor on the expression of pro-inflammatory cytokines and matrix metalloproteinases (MMPs). RA FLSs were treated with TNF-α (10 ng/ml) for 12 h in the presence or absence of AICAR (1 mM) or Compound C (Comp C, 1 µM). mRNA expression was determined by quantitative real-time PCR. **(B)** Effect of AMPK inhibition on the expression of pro-inflammatory cytokines and MMPs. RA FLSs transfected with scramble (scr) or glycogen synthase 1 (GYS1) shRNA (shG) and AMPKα shRNA (shA) were treated with TNF-α (10 ng/ml) for 24 h in the presence or absence of Compound C (Comp C, 1 µM) or AICAR (1 mM). **(C)** Effect of AMPK activation or inhibition on the proliferation of RA FLSs. Cell proliferation was determined by EdU assay. The data are presented as the mean ± SD of five independent experiments. **(D,E)** Effect of AMPK activation or inhibition on the migration **(D)** and invasion **(E)** of RA FLSs. Cells transfected or not transfected with scramble control (Scr) or GYS1 shRNA (shG) or AMPKα shRNA (shA) were serum starved overnight, seeded in a Boyden chamber, allowed to migrate for 8 h, fixed, and stained with crystal violet. TNF-α (10 ng/ml) was used as a chemoattractant. An *in vitro* invasion assay was performed using inserts coated with a Matrigel basement membrane matrix in Boyden chambers. The data are presented as the mean ± SD of five independent experiments. *P < 0.05, **P < 0.01 vs. DMSO or scramble; ^#^*P* < 0.05, ^##^*P* < 0.01 vs. TNF-α or scr + TNF-α or shG; ^&^*P* < 0.05 vs. shG + TNF-α.

To evaluate the role of AMPK in regulating synovial inflammation, RA FLSs were treated with the AMPK agonist AICAR or inhibitor Compound C. Treatment with AICAR alone reduced the expression of IL-1β, IL-6, IL-8, and MMP-9 in TNFα-induced RA FLSs (Figure 4A), and Compound C had opposite effects. Next, we determine if AMPK inhibition or AMPKα knockdown reverses the inhibitory effect of GYS1 knockdown on the expression of pro-inflammatory cytokines and MMPs. GYS1 shRNA transfection decreased the TNF-α-induced gene expression of IL-1β, IL-6, CCL-2, MMP-1, and MMP-9 compared with scramble shRNA transfection, and these effects were profound by treatment with AICAR; however, these inhibitory effects were reversed by treatment with Compound C or knockdown of AMPKα (Figure 4B). Treatment with AICAR also inhibited the proliferation, migration, and invasion of RA FLSs (Figures 4C–E). Compound C treatment or knockdown of AMPKα reversed the inhibitory effects of GYS1 knockdown on proliferation, migration, and invasion (Figures 4C–E). These results suggest that GYS1-mediated glycogen accumulation modulates inflammatory responses and the migration of RA FLSs by regulating AMPK activity.

### HIF-1α Mediates TNF-α and Hypoxia-Induced GYS1 Expression in RA FLS

**Figure 5:**
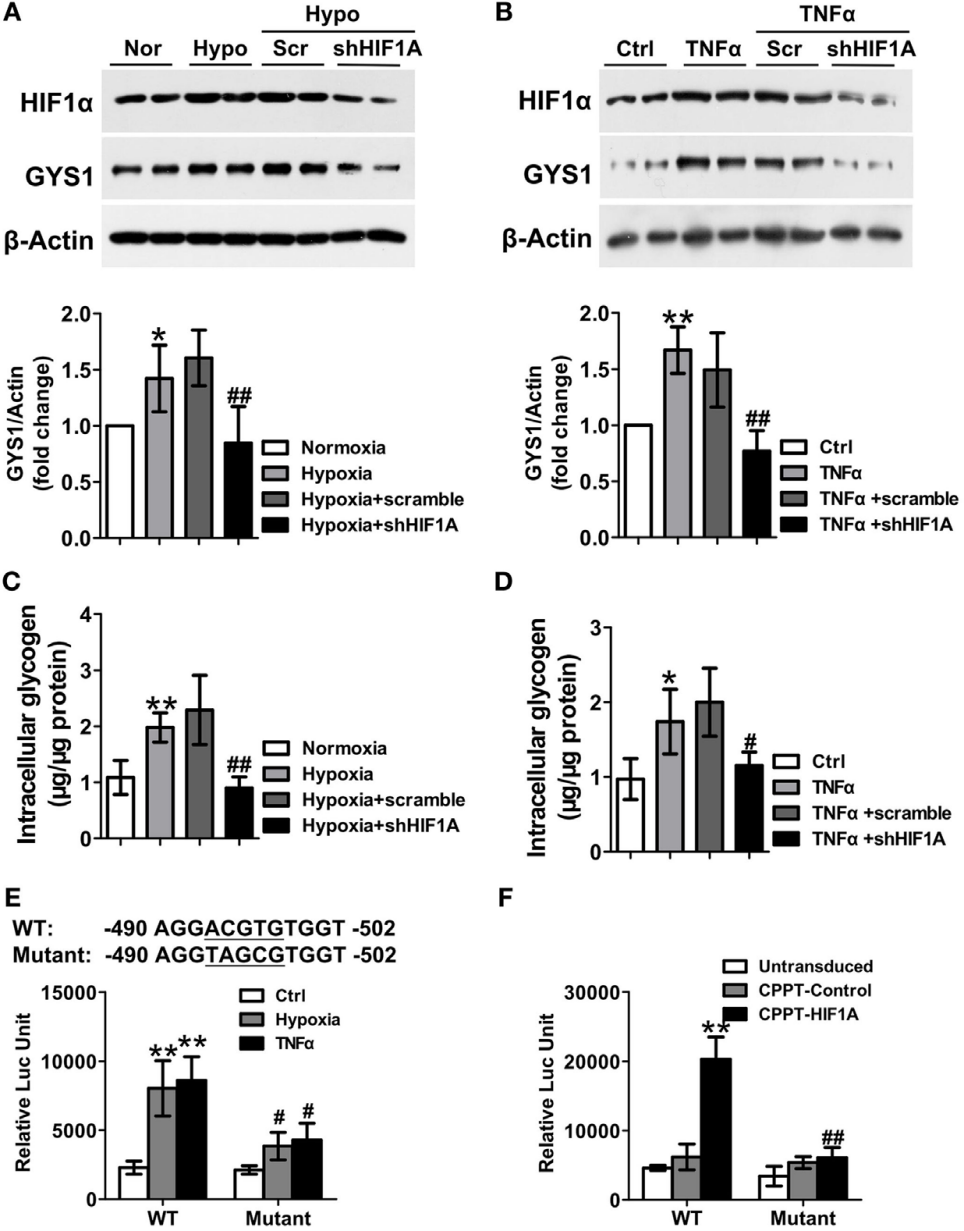
Hypoxia-inducible factor (HIF)-1α mediates the increased expression of glycogen synthase 1 (GYS1) in rheumatoid arthritis (RA) fibroblast-like synoviocytes (FLSs). **(A–D)** Effect of HIF-1α depletion on GYS1 expression and intracellular glycogen levels in RA FLSs. RA FLSs transfected with scramble or HIF1A shRNA were exposed to normoxic (21% O_2_) or hypoxic conditions (1% O_2_) **(A,C)** or stimulated with TNF-α (10 ng/ml) **(B,D)** for 24 h. The data are presented as the mean ± SEM of four independent experiments. **(E)** MH7A cells were transfected with wild-type (WT) or mutant GYS1 reporter plasmids. Approximately 48 h after transfection, the cells were exposed to 1% O_2_ or TNF-α (10 ng/ml) for 24 h, and luciferase activity was measured. **(F)** MH7A cells were co-transfected with GYS1 reporter and HIF-1α expression plasmid, and luciferase activity was measured 48 h after transfection. The data are presented as the mean ± SD of three independent experiments. *P < 0.05, **P < 0.01 vs. healthy control or normoxia or control or CPPT-Control; ^#^*P* < 0.05, ^##^*P* < 0.01 vs. osteoarthritis or hypoxia + scramble or TNF-α + scramble or WT.

Hypoxia-inducible factor-1α can induce glycogen synthesis and GYS1 expression in cancer cells (10, 11). Therefore, to define the mechanisms by which TNF-α or hypoxia promotes synovial GYS1 expression, we determined the contribution of HIF-1α to TNF-α- or hypoxia-induced GYS1 expression and glycogen synthesis in RA FLSs. First, we observed increased expression of the HIF1A gene and HIF-1α protein in RA FLSs compared with HC FLSs and OA FLSs (Figures S5A,B in Supplementary Material). In addition, HIF-1α depletion decreased the expression of GYS1 mRNA and protein (Figures 5A,B) and intracellular glycogen levels (Figures 5C,D) in hypoxia- or TNF-α-induced RA FLSs. Furthermore, to investigate how HIF-1α modulates GYS1 expression, we examined the promoter region of human GYS1 for a potential HIF-1α binding site and identified a canonical ACGTG motif at position −494. To evaluate the functionality of this binding site, we cloned the entire promoter region of the human GYS1 gene, including the putative binding site (+30 to −830), in the pGL3-basic plasmid. The promoter activity of this construct was robustly induced by hypoxia and TNF-α stimulation, but this induction was lost when the reporter construct included a mutation in the HIF-1α-binding site (Figure 5E). In addition, ectopic expression of HIF-1α remarkably increased GYS1 reporter activity in MH7A cells, a cell line of rheumatoid FLSs; however, mutation of the HIF-1α binding site completely abrogated this increased effect (Figure 5F). These findings suggest that increased rheumatoid synovial GYS1 production and glycogen levels are likely HIF-1α dependent.

### Amelioration of CIA by Local Joint Depletion of GYS1 or Intraperitoneal Administration of an AMPK Agonist

**Figure 6:**
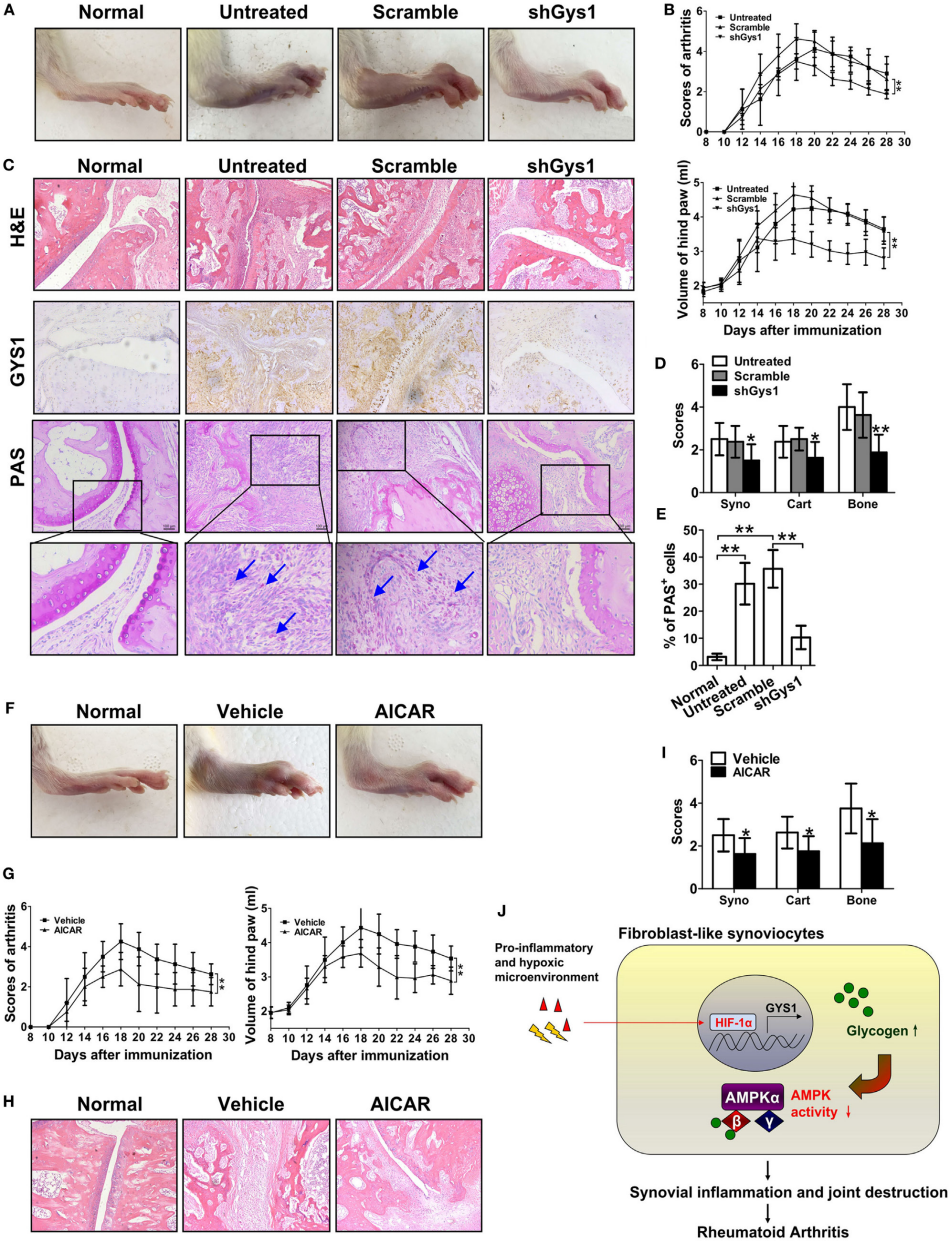
Attenuation of the severity of disease in rats with collagen-induced arthritis (CIA) by local joint depletion of glycogen synthase 1 (GYS1) or intraperitoneal administration of an AMP-activated protein kinase (AMPK) agonist. The CIA model was generated in SD rats. **(A,B)** Effect of local joint depletion of GYS1 on the articular index [upper panel of **(B)**] and paw swelling [change in volume, lower panel of **(B)**] in rats with CIA. The values in **(B)** are the mean ± SD from eight rats untreated or treated with GYS1 or scramble interference lentivirus in local joints. **(C)** Representative images of histological findings [hematoxylin and eosin (H&E), 200×], GYS1 expression (immunohistochemical staining, 200×), and glycogen [periodic acid Schiff (PAS) staining, 200×] in joint synovial tissue (ST) from CIA rats. **(D)** The scores (mean ± SD) for synovial infiltration (syno), cartilage erosion (cart), and bone loss (bone). **(E)** Semi-quantitative chart (mean ± SD) of PAS staining for ST sections from CIA rats. **(F,G)** Effect of the AMPK agonist AICAR on the severity of disease in rats with CIA. CIA rats were treated with intraperitoneal injection of AICAR (25 mg/kg/day, once daily) or 5% DMSO (vehicle, once daily) for a total of 14 days. The articular index [left panel of **(G)**] and paw swelling [change in volume, right panel of **(G)**] in rats with CIA are shown. **(H)** Representative H&E staining images and **(I)** the scores (mean ± SD) for synovial infiltration (syno), cartilage erosion (cart), and bone loss (bone) for joint ST from CIA rats. Original magnification 200×. *P < 0.05, **P < 0.01 vs. scramble or vehicle. **(J)** A model for the role of GYS1 in regulation of synovial inflammation in patients with rheumatoid arthritis.

To explore the possible *in vivo* functions of GYS1, we locally depleted GYS1 in joint tissues *via* intra-articular injection of Gys1 shRNA adenovirus in SD rats with CIA. As shown in Figures 6A,B, compared with normal rats, the arthritis index score and paw swelling (change in volume) were markedly increased in rats with CIA, whereas in GYS1-depleted rats, the arthritis index score and paw swelling were significantly reduced. Immunohistochemistry and PAS staining of joint sections revealed that GYS1 shRNA injection markedly reduced the elevated levels of GYS1 and glycogen induced by CIA in joint tissue (Figure 6C). Moreover, local deletion of GYS1 and glycogen content in joint tissues by GYS1 shRNA injection effectively inhibited synovitis and synovial hyperplasia, pannus formation and invasion into calcified cartilage and bone (Figures 6D,E). These results suggest that local joint depletion of GYS1 ameliorates the severity of experimental RA.

Furthermore, to evaluate the *in vivo* effect of AMPK, rats with CIA were treated with the selective AMPK agonist AICAR (25 mg/kg/day). Intraperitoneal injection of AICAR suppressed the increase in the arthritis index score and paw swelling compared with treatment with DMSO (Figures 6F,G). AICAR administration also decreased the inflammatory cell infiltrate and synovial hyperplasia, along with pannus invasion into calcified cartilage and bone (Figures 6H,I).

## Discussion

In this work, we demonstrated that glycogen synthesis and GYS1 expression are significantly increased in the ST of RA patients and rats with CIA and that GYS1 knockdown by shRNA inhibits the expression of pro-inflammatory cytokines and MMPs and the proliferation, migration, and invasion of cells by increasing AMPK activity. Furthermore, local joint depletion of GYS1 and treatment with AMPK agonist or knockdown AMPK markedly reduced the severity of synovial inflammation in rats with CIA. Therefore, we determined that GYS1-mediated glycogen accumulation in synovial inflammation is regulated by the suppression of excessive AMPK activity in RA, providing novel evidence that abnormal glycogen synthesis contributes to chronic inflammation.

High levels of glycogen have been observed in several tumor cell lines, including breast, kidney, bladder, uterus, skin, ovary, and leukemia cells (17, 25). Abnormal glycogen metabolism is functionally associated with the proliferation of tumor cells and poor survival in some tumors (17, 24, 25, 29). However, it remains unclear whether glycogen metabolism is involved in regulating the inflammatory response and chronic inflammatory diseases, particularly RA. In this work, we observed increased glycogen content in FLSs and ST from patients with RA compared with OA patients and HC subjects. Intracellular glycogen content represents a balance of the synthesis and degradation of glycogen, which is regulated by a series of glycogen metabolism-associated genes. Therefore, to investigate the contribution of glycogen accumulation in FLSs in RA patients, we determined the expression of all of glycogen metabolism-associated genes in FLSs from RA, OA, and HC. Interestingly, among these genes, only GYS1 gene expression was significantly increased in RA FLSs compared to OA FLSs and HC FLSs, and GYS1 protein expression was also increased in FLSs and ST from RA patients, suggesting that GYS1-mediated glycogen synthesis may result in glycogen accumulation in RA FLSs. Local synovial microenvironmental factors, particularly pro-inflammatory cytokines and hypoxia, critically influence the pathological behaviors of RA FLSs. Our study demonstrates that exposure to TNF-α or hypoxia can lead to elevated GYS1 expression and intracellular glycogen levels in RA FLSs, suggesting that increased GYS1-mediated glycogen synthesis is associated with the abnormal synovial microenvironment of RA. Glycogen accumulation and increased GYS1 expression under a hypoxic microenvironment have also been observed in some cancer and noncancer cell lines (10–12, 20); however, it is unclear if inflammatory conditions affect cellular glycogen synthesis. Therefore, our data also provide new evidence that intracellular glycogen metabolism may be driven by inflammatory factors. More importantly, we observed that depletion of the anabolic enzyme GYS1 resulted in decreased glycogen levels in association with reduced expression of pro-inflammatory cytokines and MMPs and the proliferation of TNFα- or hypoxia-induced RA FLSs. Treatment with glucocorticoid or MTX, both important anti-inflammatory agents in RA treatment, also inhibited GYS1 expression and glycogen synthesis. These results suggest that GYS1-mediated glycogen accumulation may contribute to FLS-mediated synovial inflammation in RA. These findings are also supported by our *in vivo* observation that local depletion of GYS1 in joint tissues attenuated the severity of arthritis of rats with CIA. Overall, these results implicate GYS1 as an important regulator of synovial inflammation of RA. This is the first study to provide direct evidence of a functional link between glycogen metabolism and chronic inflammatory diseases.

AMP-activated protein kinase is a heterotrimeric serine/threonine kinase containing three subunits: the catalytic α subunit and two regulatory (β and γ) subunits, and expression of these subunits varies between tissue types. AMPK not only monitors immediate energy availability by sensing AMP/ATP but may also act as a glycogen sensor *via* the glycogen-binding domain in its β-subunit (23). The significance and biological consequences of this interaction remain unclear. However, studies are increasingly indicating that, in addition to the modulation of energy homeostasis, AMPK is involved in regulating the inflammatory response (30–32) and anti-inflammatory function of AMPK activators in response to pro-inflammatory stimuli (33–36). This prompted us to investigate the regulatory role of the AMPK pathway in GYS1-mediated rheumatoid synovial inflammation. We observed reduced AMPK activity in RA FLSs compared with OA FLSs and HC FLSs, a pattern opposite that of GYS1 expression. We also observed that GYS1 knockdown increased the activation of AMPK in hypoxia- or TNFα-induced RA FLSs. These results implicate GYS1 in regulating AMPK activity in RA FLSs, consistent with a recent study that GYS1 modulates AMPK activity in leukemic cells. We also observed that AMPKβ mutation could reverse the decreased expression of phosphorylated AMPKα induced by GPI, which suggest glycogen directly inhibits the enzyme activity of AMPK by binding to AMPKβ (25). Although it was reported previously that AMPK activation affects GYS1 expression and glycogen synthesis in tumor cells (25), our data indicate that increased or reduced phosphorylation of AMPK does not result in alteration of intracellular glycogen levels or GYS1 expression in RA FLSs, suggesting a profound cell type-specific interaction of AMPK and glycogen. Taken together, our data suggest that GYS1-mediated glycogen accumulation regulates synovial inflammation by blocking the activation of AMPK. A model for the role of GYS1 in regulation of rheumatoid synovial inflammation and joint destruction is proposed in (Figure 6J).

A large body of evidence indicates that systemic anti-inflammatory and immunosuppressive therapy might not be sufficient to completely control joint destruction in RA; therefore, direct targeting of FLSs may be a new therapeutic strategy for preventing the destructive progress of RA. In this study, GYS1 knockdown blocked RA FLS activity *in vitro*, and local joint depletion of GYS1 suppressed synovial inflammation and joint destruction in rats with CIA. Similar to our results, GYS1 knockdown also reduced the tumor growth of leukemia cells transplanted in SCID mice (25). Data from mice in which the GYS1 gene is depleted indicate that targeting this protein with small-molecule agents would have little to no adverse effect in adults (37, 38). These findings suggest that directly targeting GYS1 in FLSs is a potentially viable option for alternative therapies aimed at modifying the course of the disorder. In addition, AMPK is of particular interest as a drug target for chronic inflammatory diseases because AMPK activity can inhibit inflammatory responses and NF-κB signaling in diverse types of cells and tissues (39–42). However, the potential role of AMPK in RA treatment remains unknown. Here, we determined that treatment with an AMPK agonist reduced the inflammatory response in hypoxia- or TNF-α-induced RA FLSs and ameliorated the severity of arthritis in rats with CIA, suggesting that AMPK is also a viable novel target for the treatment of RA.

In addition, a prominent characteristic of the inflamed ST of RA is hypoxia (43), indicating a possible role of HIFs in RA pathogenesis. HIF-1α is also regulated by inflammatory stimuli, including TNF-α (44). Increased HIF-1α in the RA synovium is associated with synovial inflammation and angiogenesis (45–47). Recent studies have also demonstrated that hypoxia promotes glycogen accumulation *via* the HIF-1α-mediated induction of GYS1 in some cell lines (11, 48). Therefore, we propose that HIF-1α may mediate TNF-α- or hypoxia-induced GYS1 expression in RA FLSs. As expected, our observations indicated that increased HIF-1α contributes to GYS1 expression and intracellular glycogen synthesis in TNF-α or hypoxia-induced RA FLSs, suggesting that synovial GYS1-mediated glycogen accumulation might depend on the HIF-1α pathway in RA, at least in part.

In summary, our findings demonstrate that GYS1-mediated glycogen accumulation plays an important role in regulating rheumatoid synovial inflammation by blocking excessive AMPK activation, providing a novel approach to understanding the mechanisms of RA. The suppression of GYS1 or the hyperactivation of AMPK may be a novel strategy to control various chronic inflammatory diseases, including RA.

## Ethics Statement

This study was carried out in accordance with the recommendations of the Declaration of Helsinki. The protocol was approved by the Medical Ethical Committee of the First Affiliated Hospital, Sun Yat-sen University. All patients provided written informed consent before participating in the study. All subjects gave written informed consent in accordance with the Declaration of Helsinki. The animal studies were authorized by the animal experimental ethics committee of the First Affiliated Hospital of Sun Yat-sen University.

## Author Contributions

HX, MS, YX, LL, XY, and GC designed research. MS, JW, YX, SZ, ML, CW, QQ, YTY, ZL, and YJY performed research. MS, JW, HX, YZ, YX, SZ, MH, QQ, HZ, GC, and XY analyzed data. HX, MS, JW, and GC wrote the manuscript. MS, JW, and YX contributed equally to this work.

## Conflict of Interest Statement

The authors declare that the research was conducted in the absence of any commercial or financial relationships that could be construed as a potential conflict of interest.

## Supplementary Material

Figure S1 Effect of dexamethasone (DXM) or methotrexate (MTX) on glycogen synthase 1 (GYS1) expression **(A)** and glycogen levels **(B)** in rheumatoid arthritis (RA) fibroblast-like synoviocytes (FLSs). RA FLSs were treated with 1 µg/ml dexamethasone (DXM) or 10 µg/ml methotrexate (MTX) for 24 h. Representative immune blot images are shown (left panel), and data from densitometric quantification (right panel) are expressed as the mean ± SD. Glycogen level was measured by colorimetric methods. **P* < 0.05, ***P* < 0.01 vs. Ctrl.

Figure S2 Effect of glycogen synthase 1 (GYS1) knockdown on rheumatoid arthritis (RA) fibroblast-like synoviocyte (FLS) function. RA FLSs were transfected with *GYS1* shRNA (shGYS1) or scramble controls. GYS1 expression **(A)** was measured by Western blot. Data from densitometric quantification (right panel) are expressed as the mean ± SD of four independent experiments. Intracellular glycogen levels **(B)** were detected by colorimetric methods. **(C,D)** Effect of GYS1 knockdown (shGYS1-1 and -3) on pro-inflammatory cytokines and matrix metalloproteinase expression induced by TNF-α **(C)** or hypoxia **(D)**. Transfected FLSs were unstimulated or stimulated with TNF-α (10 ng/ml) or cultured under normoxic (21% O_2_) or hypoxic conditions (1% O_2_) for 24 h. mRNA expression was determined by quantitative real-time PCR. The data are presented as the mean ± SD of four independent experiments. **(E)** Cell proliferation was determined by EdU assay. Data are presented as the mean ± SD of four independent experiments. **(F,G)** Effect of GYS1 knockdown (shGYS1-1 and -3) on the migration **(F)** and invasion **(G)** of rheumatoid arthritis FLSs. Cells transfected with GYS1 shRNA (shGYS1-1 and -3) or scramble were serum starved overnight, seeded in a Boyden chamber, allowed to migrate for 8 h, fixed, and stained with crystal violet. TNF-α was used as a chemoattractant. An *in vitro* invasion assay was performed using inserts coated with a Matrigel basement membrane matrix in Boyden chambers. The data are presented as the mean ± SD of four independent experiments. **P* < 0.05, ***P* < 0.01 vs. scramble; ^#^*P* < 0.05 vs. scramble + TNF-α or scramble + hypoxia.

Figure S3 Effect of dexamethasone (DXM) or methotrexate (MTX) on AMP-activated protein kinase activity. Rheumatoid arthritis fibroblast-like synoviocytes (FLSs) were treated with 1 µg/ml DXM or 10 µg/ml MTX for 24 h. Representative images of immune blots are shown (left panel), and data from densitometric quantification (right panel) are expressed as the mean ± SD of three independent experiments. ***P* < 0.01 vs. Ctrl.

Figure S4 AMP-activated protein kinase (AMPK)β mRNA expression in synovial tissues. Representative expression of AMPKβ1 (PRKAB1) and AMPKβ2 (PRKAB2) from healthy control (*n* = 6) and patients with osteoarthritis (*n* = 6) and rheumatoid arthritis (*n* = 6).

Figure S5 Increased expression of hypoxia-inducible factor (HIF)-1α in fibroblast-like synoviocyte (FLS) from rheumatoid arthritis (RA) patients. **(A)** Expression of the *HIF1A* gene in FLSs. Gene expression was analyzed by quantitative real-time PCR. The data are presented as the mean ± SD from healthy control (HC) (*n* = 5) and patients with osteoarthritis (OA) (*n* = 6) and RA (*n* = 9). **(B)** Protein expression of HIF-1α in FLSs. HIF-1α expression was detected by Western blot analysis. Representative images of immune blot (left panel) and densitometric quantification (mean ± SD, right panel) from HC (*n* = 5) and patients with OA (*n* = 6) and RA (*n* = 9). **P* < 0.05, ***P* < 0.01 vs. HC or OA.
