# Supplementary material for: Glycogen Metabolism and Rheumatoid Arthritis: The Role of Glycogen Synthase 1 in Regulation of Synovial Inflammation via Blocking AMP-Activated Protein Kinase Activation
Source: Front Immunol. 2018 Jul 27;9:1714. doi: 10.3389/fimmu.2018.01714 (PMC6072843; doi:10.3389/fimmu.2018.01714)
Supplement: Supplementary file 6 [file table_1.docx]

**Table S1: The sequences of shRNA oligonucleotides used in this study**

| shGYS1-1 | Forward | AATTCAAGGGCTGCAAGGTGTATTTCTCGAGAAATACACCTTGCAGCCCTTGTTTTTAT |
| --- | --- | --- |
|  | Reverse | AAAAACAAGGGCTGCAAGGTGTATTTCTCGAGAAATACACCTTGCAGCCCTTG |
| shGYS1-2 | Forward | AATTCCGCTATGAGTTCTCCAACAACTCGAGTTGTTGGAGAACTCATAGCGGTTTTTAT |
|  | Reverse | AAAAACCGCTATGAGTTCTCCAACAACTCGAGTTGTTGGAGAACTCATAGCGG |
| shGYS1-3 | Forward | AATTCATTGGCTCGGCTCAACTATCCTCGAGGATAGTTGAGCCGAGCCAATGTTTTTAT |
|  | Reverse | AAAAACATTGGCTCGGCTCAACTATCCTCGAGGATAGTTGAGCCGAGCCAATG |
| shPRKAA1-1 | Forward | AATTGTTGCCTACCATCTCATAATACTCGAGTATTATGAGATGGTAGGCAACTTTTTAT |
|  | Reverse | AAAAAGTTGCCTACCATCTCATAATACTCGAGTATTATGAGATGGTAGGCAAC |
| shPRKAA1-2 | Forward | AATTCCATCCTGAAAGAGTACCATTCTCGAGAATGGTACTCTTTCAGGATGGTTTTTAT |
|  | Reverse | AAAAACCATCCTGAAAGAGTACCATTCTCGAGAATGGTACTCTTTCAGGATGG |
| shPRKAA1-3 | Forward | AATTGCACAGACAATTGCAGTAAATCTCGAGATTTACTGCAATTGTCTGTGCTTTTTTAT |
|  | Reverse | AAAAAAGCACAGACAATTGCAGTAAATCTCGAGATTTACTGCAATTGTCTGTGC |
| shHIF1A-1 | Forward | AATTCCGCTGGAGACACAATCATATCTCGAGATATGATTGTGTCTCCAGCGGTTTTTAT |
|  | Reverse | AAAAACCGCTGGAGACACAATCATATCTCGAGATATGATTGTGTCTCCAGCGG |
| shHIF1A-2 | Forward | AATTTGCTCTTTGTGGTTGGATCTACTCGAGTAGATCCAACCACAAAGAGCATTTTTAT |
|  | Reverse | AAAAATGCTCTTTGTGGTTGGATCTACTCGAGTAGATCCAACCACAAAGAGCA |
| shHIF1A-3 | Forward | AATTGTGATGAAAGAATTACCGAATCTCGAGATTCGGTAATTCTTTCATCACTTTTTAT |
|  | Reverse | AAAAAGTGATGAAAGAATTACCGAATCTCGAGATTCGGTAATTCTTTCATCAC |
| shGys1 | Forward | AATTCCAACAAGGGAGCTGATATATCTCGAGATATATCAGCTCCCTTGTTGGTTTTTAT |
|  | Reverse | AAAAACCAACAAGGGAGCTGATATATCTCGAGATATATCAGCTCCCTTGTTGG |
| Scramble | Forward | AATTCCTAAGGTTAAGTCGCCCTCGCTCGAGCGAGGGCGACTTAACCTTAGGTTTTTAT |
|  | Reverse | AAAAACCTAAGGTTAAGTCGCCCTCGCTCGAGCGAGGGCGACTTAACCTTAGG |
