# Supplementary material for: Glycogen Metabolism and Rheumatoid Arthritis: The Role of Glycogen Synthase 1 in Regulation of Synovial Inflammation via Blocking AMP-Activated Protein Kinase Activation
Source: Front Immunol. 2018 Jul 27;9:1714. doi: 10.3389/fimmu.2018.01714 (PMC6072843; doi:10.3389/fimmu.2018.01714)
Supplement: Supplementary file 7 [file table_2.docx]

**Table S2: Sequences of primers used in this study**

| Gene | Sense | Anti-sense | Product size |
| --- | --- | --- | --- |
| GYS1 | CAGACAGTGGTTGCCTTCTTC | TTCCTCCCGAACTTTTCCTT | 131 |
| GYS2 | GTGGAACAGTGTGAACCTGTAA | AGGACTTCCTTCTATCAGCCAT | 108 |
| PYGL | CAGCCTATGGATACGGCATTC | CGGTGTTGGTGTGTTCTACTTT | 177 |
| PYGB | ACCCCAAGCGCATTTATTATCTT | GGCTTCATCGCAGGCATTC | 101 |
| PYGM | GTCCACATCAACCCCAACTC | CCCTCCAATCATCACAGTCC | 159 |
| GBE1 | GGAGATCGACCCGTACTTGAA | ACATCTGTGGACGCCAAATGA | 148 |
| HIF1A | ATCCATGTGACCATGAGGAAATG | TCGGCTAGTTAGGGTACACTTC | 125 |
| IL1B | ATGATGGCTTATTACAGTGGCAA | GTCGGAGATTCGTAGCTGGA | 132 |
| IL6 | ACTCACCTCTTCAGAACGAATTG | CCATCTTTGGAAGGTTCAGGTTG | 149 |
| IL8 | ACTGAGAGTGATTGAGAGTGGAC | AACCCTCTGCACCCAGTTTTC | 112 |
| IFNG | TCGGTAACTGACTTGAATGTCCA | TCGCTTCCCTGTTTTAGCTGC | 93 |
| CCL2 | CAGCCAGATGCAATCAATGCC | TGGAATCCTGAACCCACTTCT | 190 |
| MMP1 | CTCTGGAGTAATGTCACACCTCT | TGTTGGTCCACCTTTCATCTTC | 199 |
| MMP3 | CGGTTCCGCCTGTCTCAAG | CGCCAAAAGTGCCTGTCTT | 206 |
| MMP9 | TGTACCGCTATGGTTACACTCG | GGCAGGGACAGTTGCTTCT | 97 |
| MMP13 | TCCTGATGTGGGTGAATACAATG | GCCATCGTGAAGTCTGGTAAAAT | 184 |
| PRKAA1 | TTGAAACCTGAAAATGTCCTGCT | GGTGAGCCACAACTTGTTCTT | 113 |
| PRKAA2 | CTGTAAGCATGGACGGGTTGA | AAATCGGCTATCTTGGCATTCA | 159 |
| PRKAB1 | CCCTTCCGAGCCCATAGTAAC | GCACTTTTGGGAATCCACCATT | 112 |
| PRKAB2 | CAGTCAGCTTGGCACAATTAAC | TCCTCAGATCGAAACGCATACA | 171 |
| 18S rRNA | GACTCAACACGGGAAACCTC | AGACAAATCGCTCCACCAAC | 120 |
